# Family Dysfunction and Cyberchondria among Chinese Adolescents: A Moderated Mediation Model

**DOI:** 10.3390/ijerph19159716

**Published:** 2022-08-07

**Authors:** Shengyingjie Liu, Huai Yang, Min Cheng, Tianchang Miao

**Affiliations:** 1 Faculty of Artificial Intelligence in Education, Central China Normal University, Wuhan 430079, China; 2 School of Nursing & Institute of Higher Education Research and Quality Evaluation, Chengdu University of Traditional Chinese Medicine, Chengdu 611137, China; 3 College of Humanities & Arts, Jiaxing Nanhu University, Jiaxing 314001, China

**Keywords:** family dysfunction, cyberchondria, health anxiety, optimism, moderated mediation

## Abstract

Cyberchondria has become a severe health problem and a significant public concern. In addition to the impacts that cyberchondria involves, individual psychological and behavioral factors have been identified. However, the role of family function and the mediating and moderating mechanisms underlying these relations are not understood well, especially among adolescents. Based on family functioning and cognitive-behavioral theory, this study sought to examine whether family dysfunction was associated with cyberchondria, and a moderated mediation model was prepared as a means of exploring whether health anxiety was a mediator of relationships between family dysfunction and cyberchondria, as well as whether optimism moderated these mediating processes. A total of 2074 Chinese adolescents (mean = 15.08 years, SD = 1.79) reported their demographic information, family dysfunction, health anxiety, optimism, and cyberchondria. The findings showed that family dysfunction was positively related to cyberchondria. Moreover, health anxiety partially mediated the relationship between family dysfunction and cyberchondria. Finally, optimism moderated the interplay among health anxiety and cyberchondria. Consistent with the expectancy-value models, this positive relationship was weaker for adolescents with a higher level of optimism. These results suggest that it is vital to simultaneously consider individual and family factors as a means of understanding adolescent cyberchondria when performing cyberchondria intervention programs.

## 1. Introduction

Many people are increasingly using the Internet to obtain information about health [1]. One survey of >12,000 individuals within 12 nations revealed that over 75% of surveyed individuals in 9 of these countries utilized the Internet to search for health-related information [2]. With the overuse of health searching, cyberchondria has become a severe health problem and a significant public concern [3,4,5]. Cyberchondria refers to excessive, repeated online searches related to personal health information, resulting in distress, anxiety, and even the inability to control their search behavior [6], and its most salient characteristics involve cognitive and affective components, such as frequent concern regarding health and repetitive or excessive online searching for health-related knowledge [7]. Cyberchondria can bring some adverse effects, including increases in disease-related fear, increased health-related anxiety, confusion due to conflicting information, disruptions of other activities, and deterioration in the doctor–patient relationship [3,8,9,10].

Previous studies have identified several predictors of cyberchondria indicating that the risk factors for cyberchondria include the ambiguity of health-related details on the Internet [11], health-related anxiety and obsessive compulsive symptoms [12], inhibitory intolerance of uncertainty [13], perfectionism trait [14], and cognitive bias [15]. In contrast, the protective factors for cyberchondria are self-esteem [16], reassurance seeking, and cognitive behavioral therapy [6]. To date, however, few studies have investigated the link between family factors and cyberchondria. Family provides the most long-term and core environment for the growth and development of individuals [17]. Family is now thought to be a sophisticated system, and researchers have increasingly discussed the role of family functioning [18,19]. Adolescents from a dysfunctional family develop a range of problematic behaviors [20], such as excessive health search behavior, to ease their fear of disease without the care of the family. It is necessary to explore whether insufficient family functioning will influence individuals’ problematic cyberchondria behavior.

Teenagers undergo rapid physical and psychological changes. Early research has indicated that children can exhibit precursors to somatoform disorders (e.g., hypochondriasis) that arise in adults when medically unexplained physical symptoms are prevalent [21,22,23]. Moreover, evidence from investigators of fear in children and adolescents has shown that teenagers have sustained health-associated anxiety and fear [24,25]. Additionally, when logging onto a health website, adolescents most often look for information about diseases that match their circumstances, which is crude for self-diagnosis without taking age, gender, lifestyle, and other subtleties into consideration [26].

In addition, the study of cyberchondria is still in its infancy, with studies providing data assessing individual rather than family-related factors associated with cyberchondria, and the associated mechanisms are poorly understood, as are factors related to the vulnerable group of adolescents. To clarify these gaps, according to the McMaster Model of Family Functioning [27], dysfunctional families often do not adequately contribute to healthy social, psychological, and physical development in children and thus elevate the risk of anxiety. We, therefore, examined the degree to which health-related anxiety may function as a mediator of the interaction between family dysfunction and cyberchondria. Furthermore, the expectancy-value models of motivation were applied to the scene that positive expectations and confidence can interact with negatively psychological factors [28]. Thus, we tested whether optimism has a moderating effect on the relationship between family dysfunction and adolescent cyberchondria, either directly or indirectly.

### 1.1. Family Dysfunction and Cyberchondria

Family dysfunction can be a vulnerability factor for cyberchondria. Family dysfunction indicates that a given family facilitates inappropriate functionality due to factors such as poor communication and problem-solving [29]. As the Family Functioning theory posits, a functional family environment is essential in order to ensure that the members of that family develop in a healthy manner at the social, psychological, and physical levels, which can impact people’s internalizing symptoms (i.e., anxiety and depression) that may lead to explicit anxious behavior [27,30], including cyberchondria. Although prior studies have not shown there to be a direct relationship between family dysfunction and adolescent cyberchondria, this notion is indirectly supported by much empirical evidence. Specifically, family functioning is a negative predictor of addiction to using a smartphone among adolescents, which is a behavioral predictor for cyberchondria, as problematic users tend to obtain more health information through the Internet and have negative cognitive with interpretation errors [31].

To put it another way, given that the first thing that happens in adolescents before cyberchondria is somatization, family dysfunction can be regarded as connected to an unorganized attachment style [32], in which physical somatization may be an appropriate, not pathologic, response to stress. When adolescents perceived such somatization symptoms, their online searches, including searches regarding potentially deadly illnesses that contain misleading, frightening, or false information [4], this can lead to a worsening of illness-related anxiety or the development of new anxiety, resulting in worsening cyberchondria [3]. One may assume that family dysfunction can predict cyberchondria. Indeed, A meta-analysis has shown that family dysfunction can predict somatoform symptoms and increase health risks [33]. However, there have been no studies directly evaluating the association between family dysfunction and cyberchondria among adolescents.

### 1.2. Mediating Role of Health Anxiety

Health anxiety refers to anxiety related to one’s well-being [34], and it can be positively related to cyberchondria. Such anxiety can span a wide spectrum from intermittent worry to debilitating anxiety [6]. The DSM-5 incorporates severe health anxiety as a somatic symptom disorder or an illness-anxiety disorder [35]. Those with health anxiety exhibit constant concern regarding the development of severe or deadly illnesses or [34,36]. The cognitive behavioral theory of severe anxiety posits that someone with elevated health anxiety is more likely to incorrectly interpret vague medical information from the Internet, health checks, and disease description information [37]. Previous empirical studies have proved this notion [1,38,39]. Meta-analysis research incorporating 20 studies and 7373 participants has shown health anxiety to be positively associated with cyberchondria with a degree of medium to high [1]. A network analysis has shown, as a particular symptom, that cyberchondria is primarily associated with health anxiety as well as problematic Internet use [38]. A cross-sectional study posited a two-way model that verified that people with health anxiety checked medical conditions through the Internet to gain reassurance and psychological comfort [16]. However, this will further increase doubt and suffering from a specific primary disease because of the short-lived comfort and reassurance but will ultimately have the opposite effect of exacerbating health anxiety [39]. Since adolescents have to overcome more physical and psychological as well as social changes compared with adults, they more often look for information about specific diseases or medical problems [26]. Meanwhile, adolescents do experience significant health anxiety [40]. Based on the above literature, it is rational to presume that health anxiety can be positively associated with cyberchondria, especially among teenagers.

Even without empirical evidence, we can presume that family dysfunction is positively related to health anxiety. The McMaster family functioning model and prior empirical research show a strong family environment and support to be an integrated system; for example, family functioning may be an essential determinant of depression/anxiety [19,27]. A fair amount of empirical research on health anxiety considers it a prognostic indicator for other anxiety disorders, which is more clinically significant in diagnosing anxiety conditions than any other variables [28,41]. Conversely, people with anxiety disorders often express concern regarding personal health and other life events, as well as interpersonal relationships [42]. Such experiences are often positively associated with dysfunctional beliefs, somatosensory amplification, and experiences relevant to hypochondriasis, which lead to an interruption in users’ activities across multiple sessions and make them preoccupied with searching for content related to medical information [4,43]. Thus, one may posit that family dysfunction is linked to anxiety experiences such as health anxiety. A longitudinal study from three waves of an extensive Canadian dataset has shown that in boys and girls in middle childhood, being exposed to a dysfunctional family environment for extended periods can promote the development of anxiety behaviors [30]. Likewise, this relationship can also be applied to sensitive indicators such as health anxiety. One can, thus, assume that family dysfunction is positively linked to health anxiety.

In sum, family dysfunction may be positively related to health anxiety, which, subsequently, may be positively associated with cyberchondria. Health anxiety may correctly serve as a mediator of this association between familial dysfunction and cyberchondria.

### 1.3. Moderating Role of Optimism

Optimism refers to the extent to which a given individual holds favorable expectations pertaining to their future life experiences [44]. It is also conceptualized as an attributional style exhibited when individuals explain the success or failure of particular experiences [45,46]. Individuals with high levels of optimism were found to prospectively develop superior subjective well-being under adverse conditions, engaging appropriate coping strategies and proactively protecting their health, which is referred to as health promotion [47]. The expectancy–value motivation model suggests that motivation can be bolstered by having positive expectations and generally appears to be an approach for those coping with a time of adversity [28]. The expectancy–confidence model of motivation further argues that confidence and doubt (among optimists who are confident about eventually reaching goals) serve as key decision-making factors with respect to the interplay between negative factors and behavior modules [44,48]. Some empirical studies have confirmed these theoretical models. For instance, Hirsch, Walker, Chang, and Lyness found optimism to be associated with a less robust relationship between anxiety symptoms and the burden of illness [48]. Hirsch, Wolford, LaLonde, Brunk, et al. found optimism to serve as a significant buffer in the relationship between adverse life events and suicide ideation and previous suicide attempts [49].

Optimism can protect individuals from cyberchondria. Compared with pessimists, optimists are more likely to set recovery goals and not focus on distress and uncomfortable symptoms [46]. For instance, adolescents may exhibit a pattern of cyberchondria because it is difficult for them to tolerate the uncertainty of using the Internet to self-diagnose or self-medicate when they have access to massive amounts of information [26,50]. Optimism, however, can help adolescents improve the neuroendocrine system and boost immunity [51] and promote a more positive mood, better adaptive coping, and more protective health-related behavior [52] and thus may lower the vicious circle of cyberchondria in response to harmful factors. Therefore, optimism may buffer the acceleration of cyberchondria due to detrimental factors, including familial dysfunction and health anxiety. That is to say, optimism may serve as a mediator of the interplay between cyberchondria and familial dysfunction in a second-stage mediation model. Moreover, how family dysfunction affects anxiety- or depression-related symptoms may further be inhibited given that social anxiety can be significantly impacted by the interplay between individual and familial factors, and optimism, as an individual characteristic, can decrease adolescent health-related anxiety [53]. Considering previous research regarding the common usage of mediation and moderation models [54,55], if health anxiety serves as a mediator of the interplay between familial dysfunction and cyberchondria, and optimism moderates the front stage or the backstage of this model, then optimism would moderate the mediator role of the associated health-related anxiety.

### 1.4. Research Objective and Hypotheses

**Figure 1:**
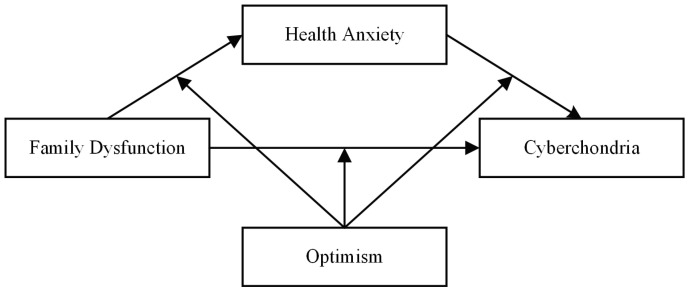
Conceptual model.

The current study constructed mechanisms underlying the association between family dysfunction and hypochondria in adolescents according to the McMaster Model of Family Functioning, in which adolescents’ positive psychological factors (optimism) and negative psychological factors (health anxiety) were integrated, which could be beneficial to enrich The McMaster Family Functioning Mode and to extend the formation mechanisms of cyberchondria. The three goals of this study were (a) to evaluate how family dysfunction affects cyberchondria; (b) to evaluate whether health anxiety is a mediator of the interplay between familial dysfunction and cyberchondria; and (c) to examine optimistic moderating effects on relationships among these variables, as mentioned above. A moderated mediation model (Figure 1) was used to shape the hypotheses guiding this study of Chinese adolescents. Specific hypotheses were as follows:

**Hypothesis** **1** **(H1).** *Family dysfunction will be positively associated with adolescent cyberchondria.*

**Hypothesis** **2** **(H2).** *Health anxiety will mediate the link between family dysfunction and adolescent cyberchondria.*

**Hypothesis** **3** **(H3).** *The direct effect of family dysfunction on adolescent cyberchondria and the mediating effect of health anxiety will be moderated by optimism, with reductions in these adverse effects among adolescents with increased optimism.*

## 2. Method

### 2.1. Participants

For this study, we recruited adolescents from two middle schools in Gansu Province, China. All the students participate in the survey. The current research included 2074 students (1024 girls, 1050 boys). The mean age of these students was 15.08 years (SD = 1.79, range = 12–20). On average, these students used the Internet 18.89 (SD = 18.67) hours per week. Sixty-five percent of participants were junior school students (1353 students), 54.2% were from rural areas, and 67.8% had no siblings. The Body Mass Index (BMI) values for 49.1% of the participants were standard. Nearly ten percent of respondents were members of non-intact families (i.e., divorced parents or both parents absent). In total, 39% of fathers and 35% of mothers had an education level of high school or greater. This information should allow us to provide a concise and precise description of the experimental results, their interpretation, as well as the experimental conclusions that can be drawn.

### 2.2. Procedure

The Ethical Committee approved all components of this study for Scientific Research at the authors’ institution. The research was conducted in two middle schools in March 2020. The Student Affairs Division of the target school was informed, and students’ consent was obtained before their participation. An online survey of adolescent students was conducted using Sojump and sent to the class group by the headteacher. Prior to the assessment, headteachers that had received specific training were tasked with instructing students regarding appropriate survey completion approaches via the use of standardized instructions, under the anonymous and confidential principle, in order to ensure that data were collected accurately and in a standardized fashion.

### 2.3. Measures

**Family Dysfunction**. Adolescents’ perception of family dysfunction at home was measured via the Family Assessment Device (FAD) [56], which incorporates nine items evaluating how much adolescents had perceived communication, mutuality, and conflict and harmony among family members (e.g., “we do not get along well”). Adolescents rated items on a four-point scale from 1 (strongly disagree) to 4 (strongly agree). Four items were scored in reverse, and the responses were then summed such that higher scores were indicative of greater family dysfunction. Prior studies have shown this questionnaire to be reliable [57]. In this study, the questionnaire had a Cronbach’s α coefficient of 0.82. In a confirmatory factor analysis (CFA), we found that our scale model exhibited good fit: χ^2^/df = 8.29, RMSEA = 0.06, CFI = 0.97, TLI = 0.96, and SRMR = 0.03.

**Cyberchondria**. Cyberchondria was assessed using a shorter iteration of the Cyberchondria Severity Scale [58]. The questionnaire includes 12 items measuring 4 aspects of cyberchondria severity: excessiveness (e.g., “If I notice an unexplained bodily sensation I will search for it on the internet”), distress (e.g., “I start to panic when I read online that a symptom I have is found in a rare/serious condition”), and reassurance (e.g., “Researching symptoms or perceived medical conditions online leads me to consult with my GP”) as well as compulsion (e.g., “Researching symptoms or perceived medical conditions online distracts me from reading news/sports/entertainment articles online”). Items were rated from 1 (never) to 5 (all the time) on a 5-point scale, after which scores corresponding to the 12 items were averaged, such that higher scores were indicative of greater cyberchondria. Previous studies have often used this questionnaire, and it has been shown to be valid and reliable among Chinese adolescent students [59]. Cronbach’s alpha in this study was 0.91, and the CFA index revealed an acceptable fit of the scale model: χ^2^/*df* = 7.92, RMSEA = 0.10, CFI = 0.93, TLI = 0.90, and SRMR = 0.04.

**Health Anxiety**. The Short Health Anxiety Inventory [28] was used as a means of assessing health anxiety unrelated to physical health status. It was later revised into the Chinese version [60]. The questionnaire includes 18 items measuring three aspects of health anxiety: “Illness Likelihood” (e.g., “Worry about health”), “Illness Severity” (e.g., “Ability to enjoy life if I have an illness”) and “Body Vigilance” (e.g., “Noticing aches and pains”). Adolescents rated items on a four-point scale from 1 (never) to 4 (always true). Two items were scored in reverse, and the 18 items’ scores were then averaged such that higher scores were indicative of a higher level of health anxiety. The questionnaire demonstrated good reliability in previous research [61]. Cronbach’s alpha for this sample was 0.82. The index of CFA showed an acceptable fit of the scale model: χ^2^/*df* = 3.85, RMSEA = 0.09, CFI = 0.92, TLI = 0.90, and SRMR = 0.05.

**Optimistic**. The Chinese version [62] of the Life Orientation Test-Revised [63] scale was used as a means of evaluating how optimistic these adolescents were. The questionnaire consists of 6 items that measured adolescents’ optimistic attitudes (“I am hopeful about my future”). Adolescents rated each item on a five-point scale from 1 (strongly disagree) to 5 (strongly agree). Three items were scored in reverse, and the six items’ scores were averaged such that higher scores were indicative of a higher level of an optimistic attitude. This tool has frequently been utilized in other studies and has been found to be reliable and valid [46,64]. In this study, this scale had a Cronbach’s α of 0.69. It is found that the scale model exhibited good fit: χ^2^/*df* = 1.17, RMSEA = 0.01, CFI = 0.99, TLI = 0.99, and SRMR = 0.01.

**Control variables**. Prior studies have shown that adolescents’ grades, family structure, socioeconomic status, and Body Mass Index (BMI), as well as online health information search, are related to cyberchondria [7,26,61,65]. These demographic variables and relevant covariates were thus controlled for in our statistical assessments, with the grade being dummy coded (0 = junior; 1 = senior), as was the family structure (0 = non-intact, 1 = intact). Socioeconomic status was measured with a standardized factor score (M = 0, SD = 1), with this score being determined based on assessments of the income, occupation, and education of both parents such that higher scores were indicative of higher family socioeconomic status [66]. Body Mass Index (i.e., BMI), a standard used to measure the degree of obesity to evaluate whether the individual is healthy or not, was measured with a mathematical formula, that is, the ratio of weight to height (i.e., weight/height ^ 2), as provided by National Health and Family Planning Commission [67]. In the following statistics, we standardized the treatment. Healthy search behavior was measured with one item (“How often do you search the Internet for information about your health?”) on a 5-point scale (1 = never, 5 = always true), with higher scores indicating more frequent health search behavior.

### 2.4. Data Analyses

**Figure 2:**
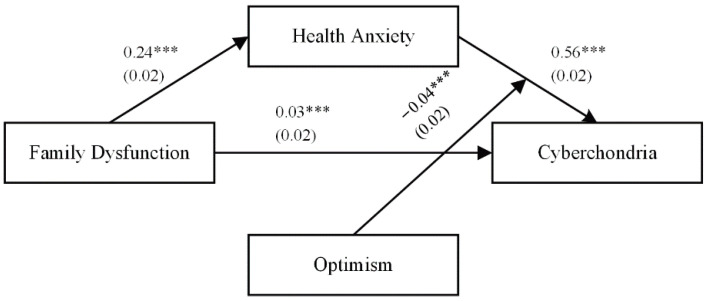
The final moderated mediation model. Note. Standardized coefficients are reported with standard errors in parentheses. Participants’ grades, family structure, SES, and BMI, as well as OHR, were controlled. *** *p* < 0.001.

Firstly, the normality of the univariate test was determined with the Harlow Test, which revealed that all the main variables were measured by univariate skewness (−1.25 < skewness < 2.0) and kurtosis (−1.0 < kurtosis < 8.0), which means that no possible outliers were found [68]. Secondly, relationships between study variables and potential covariates were evaluated using descriptive analyses. Ultimately, the hypothesized model (Figure 2) was tested using the SPSS PROCESS macro from Hayes (2018) [69]. PROCESS provides a bootstrapping estimation method for direct/indirect mediation effects, and even moderated mediation models, using 5000 random samples and put-back. For the results, when the confidence intervals (CIs) do not contain 0, then the indirect effect can be considered to be established. We initially tested whether adolescent cyberchondria was positively associated with family dysfunction (Hypothesis 1) and mediated by health anxiety (Hypothesis 2) by PROCESS (Model 4). How optimism moderates the association between family dysfunction and cyberchondria via/not via health anxiety was assessed with the PROCESS macro (Model 59) (Hypothesis 3). Simple slopes were then plotted to probe for significant interactions associated with low/high optimism levels [69]. Covariates were controlled for during all the analyses as the variables were standardized in moderated mediation analyses.

## 3. Results

### 3.1. Common Method Bias

All variables were measured by the questionnaires in this study, which can have a common method bias. Harman’s single-factor test was conducted as a statistical remedy to assess the common method bias that may have affected the true correlations between variables and caused biased parameter estimates [70]. The test results indicated that the first factor accounted for 20.90% (<40%) of the total variance; thus, there is no evidence of a substantial common method bias in this study.

### 3.2. Preliminary Analyses

The means, standard deviations, and *t*-tests of demographic variables are shown in Table 1. Differences in No gender, Only Kid, and Residence existed in cyberchondria, but there were significant gender and residence differences in family dysfunction, health anxiety, and optimism. The results revealed that girls reported more health anxiety and optimism, t (1024) = 1.97, *p* < 0.05. Furthermore, urban students reported more serious family dysfunction than rural students, t (950) = 2.19, *p* < 0.05.

Means and other related data for study variables are shown in Table 2, including bivariate correlations across all study variables, which were correlated in the expected directions. Specifically, family dysfunction and health anxiety were positively associated with cyberchondria (r = 0.15, *p* < 0.001 and r = 0.68, *p* < 0.001, respectively). That is to say, those with more serious family dysfunction were more likely to engage in cyberchondria. Therefore, H1 was supported. Moreover, optimism was negatively correlated with cyberchondria (r = −0.17, *p* < 0.001). In addition, family dysfunction was positively related to health anxiety (r = 0.24, *p* < 0.001). Finally, there was a negative correlation between optimism and health anxiety (r = −0.23, *p* < 0.001).

### 3.3. Testing for Mediation (Hypothesis 2)

H2 proposed that health anxiety would serve as a mediator of the association between family dysfunction and cyberchondria. We employed the PROCESS macro model 4 [69] in order to explore such potential mediation. The results of the mediation models are shown in Table 3, with the results (see Model 1) demonstrating that after controlling for covariates, family dysfunction positively predicted health anxiety (β = 0.24, *p* < 0.001). In other words, adolescents exposed to a less functional family environment were also likely to exhibit increased health anxiety. The Model 2 results further showed that health anxiety positively predicted cyberchondria (β = 0.56, *p* < 0.001). Adolescents with greater health anxiety were thus more likely to suffer from cyberchondria at some point in time. Additionally, after controlling for the covariates, family dysfunction was found to directly predict cyberchondria (β = 0.03, *p* < 0.05; see Model 2). Bootstrapping analyses showed that the indirect effects of family dysfunction on cyberchondria (β = 0.14, 95% CI = [0.11, 0.16]) through health anxiety were significant. This indicates that adolescents suffering from more serious family dysfunction are susceptible to increased health anxiety, placing them at an elevated risk of cyberchondria. Approximately 81% of the total effect was accounted for by this mediation effect, thus supporting H2.

### 3.4. Testing for Moderated Mediation (Hypotheses 3)

H3 assumed that optimism functioned to moderate the interplay between family dysfunction and cyberchondria via health anxiety. We examined the moderated mediation analysis with PROCESS macro (Model 59) to obtain bootstrapped confidence intervals for the mediation model at different levels of optimism [69]. In Model 1, we accurately assessed the function of optimism as a moderator of the interplay between family dysfunction and health anxiety. In Model 2, optimism functions as a moderator of the interplay between health anxiety and cyberchondria, in addition to a direct link between family dysfunction and cyberchondria. The results of the analysis are presented in Table 4.

**Figure 3:**
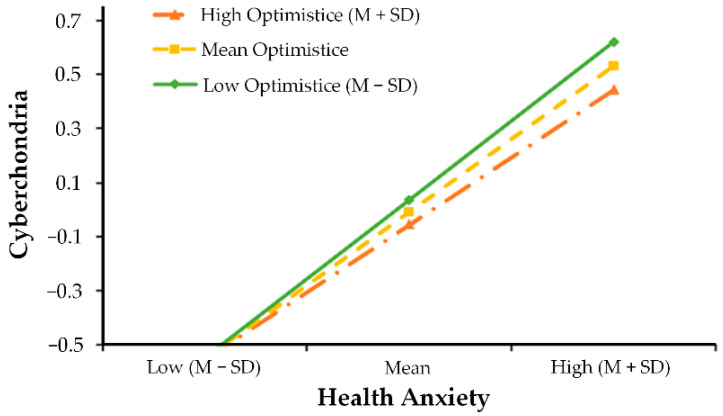
Optimistic moderated the relationship between health anxiety and cyberchondria.

As shown in Table 4, after controlling for covariates, Model 1 indicated that family dysfunction positively predicted health anxiety (β = 0.18, *p* < 0.001). Moreover, optimism (β = −0.17, *p* < 0.001) negatively predicted health anxiety. However, optimism failed to function as a significant moderator in the first half of the mediation model (i.e., family dysfunction → health anxiety; see β = −0.02, *p* = 0.16). The full model accounted for 19% of the variance in health anxiety (R^2^ = 0.19, *p* < 0.001). Likewise, Model 2 suggested that optimism did not moderate the direct links between family dysfunction and cyberchondria (i.e., s family dysfunction → cyberchondria; see β = −0.01, *p* = 0.62), but health anxiety interacted with optimism in predicting cyberchondria in the second half of the mediation model ((i.e., health anxiety → cyberchondria; see β = −0.04, *p* < 0.01). The full model accounted for 56% of the variance in cyberchondria (R^2^ = 0.56, *p* < 0.001), which shows moderate to substantial explanatory ability [71]. In order to intuitively understand the moderating effect, the relationship between health anxiety and cyberchondria was plotted across low (i.e., one standard deviation below the mean) and high (i.e., one standard deviation above the mean) levels of optimism in Figure 3. Simple slope analyses revealed that the effect of health anxiety and cyberchondria was positive and significant among lower levels of optimism (βsimple = 0.58, *p* < 0.001), but the influence slope becomes gentler among higher levels of optimism (βsimple = 0.50, *p* < 0.001). Lastly, we analyzed conditional indirect effects. These findings showed that optimism was a significant moderator of the indirect pathway from family dysfunction to cyberchondria among adolescents (Table 5). For adolescents with low optimism (1 SD below the mean), family dysfunction had a strong effect on cyberchondria through the mediating effect of health anxiety (β = 0.12, CI = [0.07, 0.16]). For individuals with high optimism (1 SD above the mean), however, there was a decrease in this mediating effect with a protective role of optimism (β = 0.08, CI = [0.05, 0.10]). Therefore, H3 was partially supported.

## 4. Discussion

Herein we employed a moderated mediation model as a means of assessing the potential correlation between family dysfunction and cyberchondria, as well as whether health anxiety would mediate the link between family dysfunction and cyberchondria and whether there would be differences in optimism between mediation process links. Our findings indicated that family dysfunction showed a positive correlation to health anxiety and adolescent cyberchondria. Furthermore, the findings suggested health anxiety mediated the link between family dysfunction and cyberchondria. Finally, the positive relationship between health anxiety and cyberchondria was weaker for those adolescent girls with higher levels of optimism. All of the findings are discussed below.

### 4.1. Family Dysfunction and Adolescent Cyberchondria

Consistent with Hypothesis 1, the present study showed that family dysfunction was positively associated with cyberchondria. While prior studies found certain individual factors for adolescent hypochondria, such as well-being, psychological distress, and health-related behavior [72], limited research has explored the link between family function and cyberchondria. Therefore, the family environment is necessary for the healthy development of the physical, psychological, and social functioning of adolescents [27,30], and a maladaptive family function makes it easy to form an unorganized attachment style, which leads to physical somatization before cyberchondria [32]. Our findings revealed the influence of adolescents’ quality of family function on their online health-related compulsive behaviors, particularly cyberchondria. This finding is in line with prior work showing that family dysfunction is significantly associated with adolescents’ online compulsive behavior and the risk of disease [73,74]. Moreover, this finding also corresponds to the family function theory (McMaster model) and prior empirical research [3,4,30], which suggest that incoherent family factors such as family dysfunction can be significant predictive factors for an individual’s somatoform symptoms and cyberchondria.

### 4.2. Mediating Role of Health Anxiety

Consistent with Hypothesis 2, we found that health anxiety mediated the link between family dysfunction and cyberchondria among adolescents. This finding verified the McMaster model and cognitive behavioral theory [27,37], which posit that health anxiety may be generated in discordant family contexts and leads to seeking reassurance from the medical advice on the Internet, and thus the misinterpretation of vague medical information. This health anxiety is temporarily alleviated through cyberchondria behavior, meanwhile, which instead exacerbates health anxiety, due to the uncertainty of medical information on the Internet. This study is the first we are aware of to extend this finding to teenage groups, and broaden prior work by revealing that family relationships can impact online hypochondriasis of adolescents via health anxiety.

In addition to the mediating effect of the model, individual links within this mediation model are worth discussing. Findings pertaining to the initial stage of this mediation process (i.e., family dysfunction → health anxiety) are congruent with the notion that problematic family functioning is a painful experience in the formation of adolescent health anxiety [19]. Adolescence is a critical life stage for the shaping of illness behavior [75]. Adolescents with high family dysfunction usually display a lower ability to communicate with family members to solve problems [76], potentially further disrupting access to sufficient support as required, resulting in the exacerbation of health anxiety [41,77]. Second, the result for the second half of the mediation model (i.e., health anxiety → cyberchondria) found that escalated health anxiety was a vulnerability factor for adolescent cyberchondria. This result aligned with previous research [1,38], which is the first study we are aware of reporting this finding among teenagers. As the cognitive-behavioral theory of severe anxiety posits, if someone has relatively high levels of health anxiety, they are more likely to seek health information and misread this information without filtering [37]. Since adolescents have to overcome more physical and psychological as well as social changes, they most often look for information about specific diseases or medical problems [26]. In this condition, as social compensation theory argues, Internet service, including self-diagnosis or seeking a doctor for medicine, provides adolescents lacking family support with an in loco parentis tool to meet their needs online [78] and assuage their fear of the unknown.

### 4.3. The Moderating Roles of Optimism

We investigated optimism as a moderator of the indirect relationship between family dysfunction and cyberchondria through health anxiety, and these associations were found to be weaker for more optimistic adolescents, partially supporting Hypothesis 3. That is to say, optimism moderates the path of health anxiety and cyberchondria, establishing the second stage of the moderation model. Our results also showed that the relationship between family dysfunction and health anxiety or cyberchondria was not moderated by optimism. These results are congruent with the expectancy–value models of motivation [28], the expectancy–confidence model [46], and related studies identifying the protective role of optimism [48,49,79]. As discussed above, a state of health anxiety is a context with a time of adversity that often leads to negative, irrational coping strategies [39], and optimism can help adolescents promote protective health behaviors, adaptive coping strategies, and the enhancement of positive mood [52], thus lowering the risk of a vicious circle of cyberchondria. Based on the expectancy–value and expectancy–confidence models, when adolescents are amid adversity, positive expectations and confidence about reaching goals can contribute to obtaining rational, effective decision-making, which guides following the behavior module [46].

In contrast to our hypothesis, optimism was not a moderator of the link between family dysfunction and adolescent cyberchondria. One possible explanation is that the family plays a crucial role in shaping personality traits, such as optimism and pessimism. If the family system does not facilitate appropriate functioning, it is quite difficult to raise an optimistic child [80]. In other words, the optimistic personality of adolescents is nested under the influence of family function. If this essential family function is not fulfilled, then people are likely to engage in anxious behaviors, such as cyberchondria, regardless of their level of optimism. Additionally, optimism did not serve as a moderator of the association between family dysfunction and health anxiety. This is also due to the strong support of family function in adolescents’ psychological development. Health anxiety can be regarded as fears that revolve around health-related physical somatization [81], in which adolescents always seek comfort from family members, while family dysfunction can be regarded as an unorganized attachment style [32], which blocks the channel of such comfort and support. Therefore, adolescents with acute family dysfunction would have a high level of health anxiety regardless of their optimism.

### 4.4. Limitations and Future Opportunities

Although this is the further study to explore cyberchondria among Chinese adolescents from an ecological perspective, it has multiple limitations. First, as this was a cross-sectional study, it is not possible to make any causal inferences. Further longitudinal and experimental research are needed to yield more rigorous results based on the timeline. Second, our data volume was not large enough, potentially limiting the ecological validity of our findings. Alternatively, future studies will require more substantial and more diverse samples. As this study was based on self-reported data, its accuracy may be affected by the potential for biases (such as a common method or biases). Nevertheless, given that adolescents are sensitive to family experiences [82], employing anonymous self-reporting to evaluate family dysfunction can eliminate reputational and social-approval biases [83]. However, further studies may try collecting data from multiple informants. Fourth, future research should consider various variables. The fact that health anxiety is only a partial mediator in our model indicated that other mediating variables (such as uncertainty avoidance) might exist.

### 4.5. Practical Implications

Despite some limitations, our study has multiple practical implications. For one, these results offer evidence that family dysfunction serves as a critical risk factor associated with cyberchondria incidence among adolescents. It is thus vital that parents be aware of this risk and make efforts to eliminate dysfunction by making families better at communicating and combatting problems [76]. It is also vital that administrators at schools pay further attention to collaborations between families and schools, particularly in cases where families are facing challenges. Our work also shows that health anxiety was a critical carrier linking family dysfunction to adolescent cyberchondria, which indicates that severe health anxiety may be a necessary intervention target. For example, the online comic-based six-lesson Health Anxiety Course is a cognitive-behavioral therapy (CBT) intervention designed to combat cyberchondria and associated health anxiety [6]. Our work further shows that optimism moderates the interplay between health anxiety and adolescent cyberchondria, which suggests that optimism is conducive to alleviating excessive health anxiety and cyberchondria. Previously studies have shown that optimism can vary in response to situational changes [84,85], which means there are effective ways to intervene in terms of optimism. For example, many researchers utilized the Best Possible Self Intervention, wherein individuals receive a combination of self-compassion training and are encouraged to visualize and develop goals for an ideal future version of themselves [86,87].

## 5. Conclusions

Overall, the present study indicated that family dysfunction showed a positive correlation to health anxiety and adolescent cyberchondria, and health anxiety mediated the link between family dysfunction and cyberchondria. Moreover, the positive relationship between health anxiety and cyberchondria was weaker for those adolescent girls with higher levels of optimism. The findings suggest that it is vital to simultaneously consider individual and family factors as a means of understanding adolescent cyberchondria when performing cyberchondria intervention programs.

## Figures and Tables

**Table 1:** Means, standard deviations, and *t*-tests of main variables.

	Total	Gender			Only Kid			Residence		
	Sample	Female	Male	*t*	Only One	More than One	*t*	Urban	Rural	*t*
	M (SD)	M (SD)	M (SD)		M (SD)	M (SD)		M (SD)	M (SD)	
FD	1.99 (0.48)	1.98 (0.49)	2.01 (0.46)	1.19	1.99 (0.47)	2.00 (0.50)	0.52	1.97 (0.49)	2.02 (0.47)	2.19 *
HA	1.86 (0.43)	1.85 (0.40)	1.88 (0.46)	1.97 *	1.86 (0.42)	1.86 (0.45)	0.04	1.86 (0.44)	1.87 (0.42)	0.54
OP	3.59 (0.58)	3.61 (0.58)	3.56 (0.57)	1.97 *	3.60 (0.57)	3.57 (0.59)	0.85	3.58 (0.58)	3.59 (0.57)	0.30
CC	2.15 (0.79)	2.12 (0.72)	2.18 (0.85)	1.87	2.14 (0.78)	2.16 (0.80)	0.53	2.16 (0.77)	2.14(0.80)	0.70

Note. N = 2074. FD = Family Dysfunction, HA = Health Anxiety, OP = Optimism, CC = Cyberchondria. Gender was dummy-coded as 0 = Female and 1 = Male; Only Kid was dummy coded with such that 0 = Only one kid and 1 = More than one kid; Residence was dummy-coded with such that 0 = Urban and 1 = Rural. * *p* < 0.05.

**Table 2:** Descriptive statistics and between-variable correlations.

Variables	M (SD)	1	2	3	4	5	6	7	8	9
1. Grade	-	-								
2. Family structure	-	−0.01	-							
3. Socioeconomic status	-	0.16 ***	0.00	-						
4. BMI	19.91 (3.43)	0.17 ***	−0.03	0.04	-					
5. OHR	2.69 (1.21)	0.00	−0.02	0.01	0.00	-				
6. FD	1.99 (0.48)	−0.03	−0.04 *	−0.13 ***	0.01	−0.01	-			
7. HA	1.86 (0.43)	0.04	−0.02	−0.03	−0.02	0.33 ***	0.24 ***	-		
8. OP	3.59 (0.58)	0.02	0.04	0.08 ***	−0.02	0.00	−0.39 ***	−0.23 ***	-	
9. CC	2.15 (0.79)	0.06 **	−0.05 *	0.03	0.02	0.52 ***	0.15 ***	0.68 ***	−0.17 ***	-

Note. N = 2074. Correlations are presented below the diagonal. BMI = Body Mass Index, OHR = Online Health Research, FD = Family Dysfunction, HA = Health Anxiety, OP = Optimism, CC = Cyberchondria. Grade was coded as 0 = Junior and 1 = Senior; Family structure was coded as 0 = non-intact family and 1 = intact family. * *p* < 0.05. ** *p* < 0.01. *** *p* < 0.001.

**Table 3:** The Mediation Effects of Family Dysfunction on Cyberchondria.

Predictors	Model 1 (HA)				Model 2 (CC)			
	β	SE	*t*	95%CI	β	SE	*t*	95% CI
Grade	0.11	0.04	2.55 *	[0.03, 0.20]	0.07	0.03	2.13 *	[0.00, 0.13]
Family structure	−0.02	0.07	−0.30	[−0.15, 0.11]	−0.10	0.05	−1.97 *	[0.20, 0.00]
Socioeconomic status	−0.01	0.02	−0.25	[−0.05, 0.04]	0.04	0.02	2.42 *	[0.01, 0.07]
BMI	−0.04	0.02	−1.76	[−0.08, 0.00]	0.02	0.02	1.34	[−0.01, 0.05]
OHR	0.33	0.02	16.59 ***	[0.29, 0.37]	0.33	0.02	21.23 ***	[0.30, 0.36]
FD	0.24	0.02	11.92 ***	[0.20, 0.28]	0.03	0.02	2.04 *	[0.01, 0.06]
HA					0.56	0.02	34.85 ***	[0.53, 0.59]
R^2^	0.17				0.56			
*F*-value	70.38 ***				372.48 ***			

Note. All continuous independent variables were standardized prior to analysis. Model 1 for Health Anxiety. Model 2 for Cyberchondria. The beta values are standardized coefficients. CI = [LLCI, ULCI], i.e., lower and upper limit of the 95% bias-corrected confidence interval. BMI = Body Mass Index, OHR = Online Health Research, FD = Family Dysfunction, HA = Health Anxiety, CC = Cyberchondria. * *p* < 0.05. *** *p* < 0.001.

**Table 4:** Testing the Moderated Mediation Effect of Family Dysfunction on Cyberchondria.

Predictors	Model 1 (HA)				Model 2 (CC)			
	β	SE	*t*	95%CI	β	SE	*t*	95%CI
Grade	0.12	0.04	2.71 **	[0.03, 0.20]	0.07	0.03	2.13 *	[0.01, 0.13]
Family structure	−0.00	0.07	−0.04	[−0.14, 0.13]	−0.10	0.05	−1.91	[−0.19, 0.00]
Socioeconomic status	0.00	0.02	0.04	[−0.04, 0.04]	0.04	0.02	2.47 *	[0.01, 0.07]
BMI	−0.04	0.02	−1.98 *	[−0.08, 0.00]	0.02	0.02	1.16	[−0.01, 0.05]
OHR	0.33	0.02	16.68 ***	[0.29, 0.37]	0.33	0.02	21.35 ***	[0.30, 0.36]
OP	−0.17	0.02	−7.72 ***	[−0.21, −0.12]	−0.04	0.02	−2.50 *	[−0.07, −0.01]
FD	0.18	0.02	8.06 ***	[0.13, 0.22]	0.02	0.02	1.05	[−0.02, 0.05]
FD × OP	−0.02	0.02	−1.41	[−0.06, 0.01]	−0.01	0.01	−0.49	[−0.03, 0.02]
HA					0.54	0.02	31.59 ***	[0.51, 0.57]
HA × OP					−0.04	0.02	−2.76 **	[−0.07, −0.01]
R^2^	0.19				0.56			
F-value	61.94 ***				263.804 ***			

Note. Before analyses, continuous independent variables were standardized. Models 1 for Health Anxiety. Model 2 for Cyberchondria. The beta values are standardized coefficients. *CI = [LLCI, ULCI]*, i.e., lower and upper limit of the 95% bias-corrected confidence interval. BMI = Body Mass Index, OHR = Online Health Research, FD = Family Dysfunction, HA = Health Anxiety, OP = Optimism, CC = Cyberchondria. * *p* < 0.05. ** *p* < 0.01. *** *p* < 0.001.

**Table 5:** Conditional indirect effect analysis at levels of the Moderator (optimism).

Level of Moderator (Optimistic)	Indirect Effect			
	β	SE	LLCI	ULCI
Low (1 standard deviation below mean)	0.12	0.02	0.07	0.16
Mean	0.09	0.01	0.07	0.12
High (1 standard deviation above mean)	0.08	0.01	0.05	0.10

Note. Confidence intervals (CIs) that do not contain 0 are significant, i.e., lower and upper limit of the 95% bias-corrected CI. SE = Bootstrapping standard error.
